# Nitrogen Requirements in Healthy Adults: A Systematic Review and Meta-Analysis of Nitrogen Balance Studies

**DOI:** 10.3390/nu17162615

**Published:** 2025-08-12

**Authors:** Daisuke Suzuki, Kohsuke Hayamizu, Chiharu Uno, Yoko Hasegawa, Masashi Kuwahata, Yasuhiro Kido, Yoshio Suzuki

**Affiliations:** 1 Department of Biological Production Science, United Graduate School of Agricultural Science, Tokyo University of Agriculture and Technology, Fuchu 183-8509, Japan; dadadada0616@gmail.com; 2 Laboratory of Food Chemistry, Yokohama University of Pharmacy, Yokohama 245-0066, Japan; k.hayamizu@hamayaku.ac.jp; 3 Graduate School of Nutritional Science, Nagoya University of Arts and Sciences, Nisshin 470-0196, Japan; chiharuu@nuas.ac.jp; 4 Department of Bio-Engineering Nursing, Graduate School of Nursing, Ishikawa Prefectural Nursing University, Kahoku 929-1210, Japan; haseyoko@ishikawa-nu.ac.jp; 5 Laboratory of Nutrition Science, Graduate School of Life and Environmental Sciences, Kyoto Prefectural University, Kyoto 606-8522, Japan; kuwahata@kpu.ac.jp; 6 Department of Nutrition, Faculty of Nutritional Science, Kanazawa Gakuin University, Kanazawa 920-1392, Japan; y-kido@kanazawa-gu.ac.jp; 7 Graduate School of Health and Sports Science, Juntendo University, Inzai 270-1695, Japan

**Keywords:** nitrogen requirement, nitrogen balance, protein, recommendation, reference value, amino acid, recommended dietary allowance, estimated average requirement, dietary reference intake (DRI)

## Abstract

**Background:** Nitrogen balance studies have traditionally been used to estimate protein requirements in adults. However, ethical and practical constraints have made new studies increasingly difficult to conduct. This systematic review and meta-analysis aimed to compile and analyze the most comprehensive individual-level dataset to date. **Methods:** We included 31 studies that reported nitrogen intake and excretion data for healthy adults. Studies were selected based on strict eligibility criteria that required data from at least three intake levels per individual. Nitrogen requirements were estimated using regression analysis. In total, data from 395 individuals were analyzed. We used a random effects model for the meta-analysis. Subgroup comparisons and meta-regression were conducted based on sex, age, climate, and protein source. **Results:** The overall mean nitrogen requirement was 104.2 mg N/kg/day. No significant differences were found by sex, age group (<60 vs. ≥60 years), climate (temperate vs. tropical), or protein source (animal, plant, or mixed). Subgroup and meta-regression analyses did not reveal consistent moderator effects. Substantial heterogeneity was observed (*I*^2^ > 90%). **Conclusions:** This analysis provides the most extensive compilation of individual-level nitrogen balance data to date. While the average nitrogen requirement was consistent with previous estimates, the high heterogeneity limits definitive conclusions. Nonetheless, this dataset provides a valuable foundation for revising protein intake recommendations and guiding future research on human nitrogen metabolism in the absence of new studies.

## 1. Introduction

The World Health Organization (WHO) and the Food and Agriculture Organization (FAO) have jointly reviewed protein requirements since the 1960s. In their 1965 report, they proposed the factorial method, which estimates nitrogen requirement for adults as 95 mg N/kg/day by combining nitrogen excreted in urine, feces, and skin, each multiplied by a coefficient, along with nitrogen accumulated in the body during growth [1]. This approach was also adopted in the 1973 report by the Joint FAO/WHO Ad Hoc Expert Committee on Energy and Protein Requirements [2]. While that report acknowledged the availability of nitrogen balance study results, it also highlighted limitations in the data [2].

In 1985, nitrogen requirements were determined based on nitrogen balance studies: the safe level for adults was 0.75 g protein/kg/day, which is equivalent to 120 mg N/kg/day of nitrogen [3]. In the 1990s, a working group from the FAO, WHO, and the United Nations University (UNU) recommended conducting a meta-analysis of available nitrogen balance data on adults, including men, women, and older adults [4]. In response, Rand et al. conducted a meta-analysis of nitrogen balance studies dating back to the 1970s: the average requirement for adults was 104.6 mg N/kg/day [5]. These recommendations were last updated in 2007. The average requirement for adults is 105 mg N/kg/day [6], and remain the most recent WHO/FAO/UNU guidance to date.

The nitrogen balance method estimates nitrogen requirements by identifying the intake level needed to achieve zero nitrogen balance, indicating equilibrium between intake and loss [3,5]. However, previous studies on obligatory nitrogen loss have shown that urinary nitrogen excretion stabilizes at a specific level after five or more days on a nitrogen-free diet [7], a finding already recognized in the 1965 WHO/FAO report [1]. The 1973 Joint FAO/WHO Ad Hoc Expert Committee on Energy and Protein Requirements report [2] also noted that data from metabolic balance studies may not reflect actual physiological requirements and that nitrogen balance may not be an adequate indicator of optimal nutritional status [8].

Additional methodological considerations were later summarized by Millward during a meeting of the Macronutrient Metabolism Group of The Nutrition Society in 2000 [9]. However, in the absence of validated and acceptable alternatives, zero nitrogen balance—which includes appropriate estimates of transdermal and miscellaneous losses—has been used as the standard criterion for determining protein intake [5], as endorsed in the 2007 WHO/FAO/UNU recommendations [6].

In the 2017 revision of protein recommendations by the German, Austrian, and Swiss Nutrition Societies (D-A-CH), a structured literature review was conducted using PubMed, covering publications from 2000 to 2017. Protein recommendations for adults were derived from nitrogen balance studies [10]. More recently, there have been calls to revise these recommended protein intake levels [11], with increasing attention given to alternative methods such as the indicator amino acid oxidation (IAAO) method [12]. However, a scoping review summarizing IAAO-based studies published up to 2023 emphasized the need for further research across diverse populations [13].

The nitrogen requirement, as determined by nitrogen balance, represents the minimum intake necessary to achieve equilibrium when individuals are adapted to a specific protein level. Consequently, it is naturally lower than the intake level required to maintain zero balance in active individuals. However, in the absence of validated and widely accepted alternatives, zero nitrogen balance remains the prevailing standard for determining protein requirements.

Nitrogen balance experiments require strict dietary management and the complete collection of all excreta, including urine and feces, to accurately determine nitrogen requirements. Participants must adhere to a low-protein diet for a minimum of 5 days—a practice that raises ethical concerns. Meanwhile, the Declaration of Helsinki has undergone several revisions—most notably in 2000 (Edinburgh), 2008 (Seoul), 2013 (Fortaleza), and most recently in 2024—with increasingly stringent requirements for evaluating research risks and benefits. Concurrently, individual countries have implemented legal reforms to align with these evolving ethical standards and technological advancements. Therefore, conducting nitrogen balance studies in humans has recently become practically unfeasible.

In 2014, Li et al. conducted a meta-analysis [14] to update Rand et al.’s meta-analysis [5], identifying only three eligible studies published after 2000 [15,16,17], with the most recent published in 2010 [17]. During the 2019 D-A-CH literature review, these two systematic reviews [5,14] were used to support the revision of protein reference values. Although future research may be limited, the results of nitrogen balance studies conducted over nearly half a century remain valuable for addressing malnutrition and related issues, as they provide critical insights into the minimum nitrogen requirement for humans.

However, the datasets used in the two previous meta-analyses [5,14] were not made publicly available. Therefore, the objective of this study was to compile a comprehensive dataset on the nitrogen requirements of adult humans by retrieving individual-level data from the meta-analyses conducted by Rand et al. in 2003 [5] and Li et al. in 2014 [14] and supplementing it with an updated literature search. Meta-analyses were also performed to confirm consistency with previous studies. The compiled dataset is then provided to facilitate further research. This systematic review did not aim to discover or present new findings. Rather, it aimed to summarize research conducted over the past half-century on nitrogen requirements in adults using the nitrogen balance method. The review is expected to serve as a foundation for future research on protein requirements.

## 2. Materials and Methods

### 2.1. Collection of the Literature

First, we selected studies from the two previous systematic reviews [5,14] that provided individual-level data on protein requirements. We also included studies cited in the 2017 revision of the D-A-CH protein recommendations [10]. After removing duplicates, we reviewed the full texts of the remaining studies and included those that met the inclusion criteria.

To ensure that none of the relevant literature was overlooked, we conducted a PubMed search on 16 May 2023, using the same search terms employed by Li et al. [14]: “(dietary Proteins[MESH] OR dietary protein? OR protein requirement? OR protein allowance OR protein intake? OR protein need?) AND (Nitrogen/metabolism[MESH] OR nitrogen balance).” Titles and abstracts of the retrieved articles were screened by two independent reviewers. Studies containing data on nitrogen balance or nitrogen requirements were identified and extracted. We then checked for duplicates with studies already included from [5,10,14]. Full texts of the remaining articles were reviewed, and eligible studies were included in the final selection.

### 2.2. Inclusion and Exclusion Criteria

We selected studies that estimated the nitrogen intake required to achieve nitrogen balance in healthy individuals. Studies were included if they met all of the following inclusion criteria and none of the exclusion criteria.

The inclusion criteria were as follows: 1. The participants were healthy adults. 2. Nitrogen balance was assessed at three or more intake levels for each individual, and the zero balance point was included within that range. 3. Intake and excretion amounts were reported, or a regression equation derived from intake and excretion data was provided. If a regression equation was available, it was prioritized. 4. The study included descriptions of miscellaneous nitrogen losses other than fecal and urinary losses, including explicit statements if such losses were not measured.

The exclusion criteria were as follows: 1. The participants were children, patients, or athletes. 2. The adaptation period prior to nitrogen balance measurement was less than 5 days.

### 2.3. Data Extraction

The following information was extracted from each study: study location and climate, participant characteristics (sex, age), protein sources, nitrogen intake, nitrogen excretion, and nitrogen balance.

Protein sources were categorized as follows: animal (milk, meat [beef, fish, etc.], and eggs; plant (rice, wheat, cottonseed, potatoes, soybeans, and other legumes); mixed (both animal and plant sources).

When the age of individual participants was not provided, the average age of the group was used. If the climate was not described, it was estimated based on the study location as described in the text. If the location was also not specified, the climate was inferred from the author’s institutional affiliation.

Physical activity and total energy intake were not extracted, as these variables were not consistently reported across studies.

### 2.4. Study Risk of Bias Assessment

The Consolidated Standards of Reporting Trials (CONSORT) statement, which serves as a guideline for reporting randomized controlled trials (RCTs), was first published in 1996 [18]. Subsequently, the Preferred Reporting Items for Systematic Reviews and Meta-Analyses (PRISMA) guidelines, originally introduced as the Quality of Reporting of Meta-Analyses (QUOROM) statement in 1999 [19], were developed to guide systematic reviews of RCTs reported according to the CONSORT guidelines.

However, many of the studies included in our review were non-randomized intervention studies published before 1996 and often lacked descriptions related to the risk of bias. Moreover, the primary objective of this study was to comprehensively extract data from a wide range of literature, rather than to evaluate the quality of individual studies. Therefore, we did not perform individual risk of bias assessments for each study. However, we assessed heterogeneity among studies as part of the meta-analysis.

### 2.5. Nitrogen Requirement

Nitrogen intake, excretion, and zero balance were standardized to units of mg N/kg/day. For miscellaneous nitrogen losses (e.g., through sweat, hair, tooth brushing, and ammonia in exhaled breath), if these were not directly measured, values were imputed as 4.8 mg N/kg/day for studies conducted in temperate regions and 11 mg N/kg/day for those in tropical regions—consistent with the two preceding systematic reviews [5,14]. However, in two studies (Study ID = 26_Egun GN & 28_Campbell in Table 1) that measured skin and menstrual losses among the miscellaneous losses, the remaining unmeasured losses were set to 1.77 mg N/kg/day, following the method used in Rand et al.’s review [5].

Using the individual-level nitrogen intake and excretion data extracted from the literature, a regression equation was derived to determine the nitrogen intake required to achieve zero nitrogen balance. This zero balance value was adopted as the nitrogen requirement, in accordance with the review by Rand et al. [5]. Additionally, when regression equations extracted from the literature were individual specific and did not account for miscellaneous losses—or when the losses were derived from the above assumptions—adjustments were made so that excretion equaled zero, and the corresponding intake was then considered the nitrogen requirement. All procedures were initially performed by one researcher (YS); these results were reviewed and corrected by three additional researchers (CU, DS, YH).

### 2.6. Statistical Analysis

The normality of the nitrogen requirement distribution was assessed using the Shapiro–Wilk test. Comparisons between two groups (climate, sex, age) were performed using the Student’s *t*-test, while comparisons among protein sources (animal, plant, mixed) were conducted using the Kruskal–Wallis test. Representative values (means and 95% confidence intervals, 95% CI) were obtained by back-transforming natural log-transformed values to their original ones. All statistical analyses were performed using IBM SPSS version 24 (IBM Japan, Tokyo, Japan), with a significance threshold set at *p* < 0.05.

Meta-analysis was conducted using Cochrane Program Review Manager (RevMan) version 5.4 and R version 3.6.1. A random effects model was applied using the generic inverse variance method to account for heterogeneity in study designs. The DerSimonian and Laird method was used to calculate pooled mean nitrogen balance values and their 95% CI. Heterogeneity among studies was evaluated using the Cochrane Q (χ^2^) test with α < 0.10 and quantified using the *I*^2^ statistic.

Subgroup analyses were performed to assess whether meta-analysis results varied by sex, climate, or protein source. Additionally, meta-regression analysis was conducted to examine the effect of age.

## 3. Results

### 3.1. Literature Selection and Data Extraction

**Figure 1:**
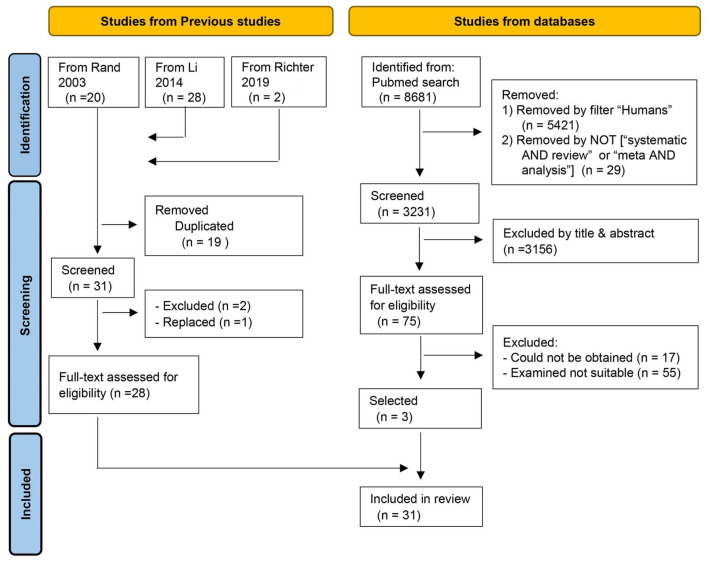
Flow diagram illustrating the literature search and selection process [5,10,14].

Twenty-eight studies were selected from the two preceding systematic reviews [5,14] and the 2017 revision of the D-A-CH protein recommendations [10]. A new PubMed search yielded three additional studies. Data were extracted from the 31 selected studies to calculate nitrogen requirements (Figure 1). The details are as follows.

#### 3.1.1. Literature Selection from Previous Studies

Table 1, “Nitrogen balance studies used to estimate protein requirement of healthy adults”, from Rand et al.’s systematic review [5] listed 21 studies categorized as “multiple intakes per individual” and “individual data published.” One study was excluded because it assessed only two intake levels. The remaining 20 studies were selected.

Among the 28 studies included in Li et al.’s systematic review [14], 27 were selected. One study [20] was unavailable; however, an alternative paper [21] was identified based on the authors, affiliation, and abstract and was judged to report the same content. Therefore, all studies included in Li et al.’s systematic review [14] were accounted for.

From the 2017 revision of the D-A-CH protein recommendation [10], we selected two studies [22,23] that stated “protein requirements may be slightly higher in older adults.” We reviewed the full texts of the 31 papers remaining after removing 19 duplicates from the literature selected using the above procedure. Three papers were excluded for the following reasons:

- One paper [15] from Li et al.’s review [14] reported individual protein requirements calculated using regression equations, with miscellaneous losses set at 8 mg/kg/day. However, nitrogen intake, excretion, and the regression equations themselves were not reported, making it impossible to adjust the requirement to 4.8 mg/kg/day based on the study site’s template climate.
- Two papers [22,23] from the 2017 D-A-CH revision [10] reported results for only single protein intake levels.
- The remaining 28 studies had their metadata extracted, nitrogen requirements obtained, and were included in this review.

At this stage, one study selected from Li et al.’s systematic review [14] measured nitrogen excretion only in feces and urine, without mention of how miscellaneous loss was set [21]. However, a table in the study summarizing average nitrogen balance data reported “skin” losses as 5.0 mg/kg/day under all conditions [21]. Therefore, we assumed that miscellaneous losses were set at 5.0 mg N/kg/day and corrected the nitrogen requirement to 4.8 mg N/kg/day.

#### 3.1.2. Literature Selection from the PubMed Search

The PubMed search was conducted on 16 May 2023, yielding 8681 papers. This was narrowed down to 3260 by filtering for “Human” studies. Subsequently, 3231 papers that were not captured by the search terms “meta-analysis” (242,208 results) or “systematic review” (225,350 results) were listed. The titles and abstracts of these papers were independently reviewed by two individuals, resulting in the selection of 75 papers that may include data on individual nitrogen balance or relevant requirements. Attempts were made to obtain the full texts of the 75 selected papers; however, 17 could not be obtained [24,25,26,27,28,29,30,31,32,33,34,35,36,37,38,39,40] (Supplementary Table S1).

After reviewing the full texts of the remaining 58 papers, three were selected. From one paper [41], nitrogen balance data were extracted to calculate the requirement. Two papers [42,43], determined to be based on the same experiment, were used in combination. Specifically, participant age and sex were extracted from one paper [42] and individual nitrogen balance regression equations were extracted from the other [43] to calculate requirements for each participant.

The data obtained from these three papers [41,42,43] were included in this review.

### 3.2. Metadata

The extracted metadata differed from the previous systematic reviews [5,14] in the following ways:

- The protein source in one study [44] was classified as “Mixed” in the 2003 review [5], but the paper states “animal protein diet”, confirming it was of animal origin.
- In another study [45], the protein source was also listed as “Mixed” in the 2003 review [5], but the paper indicates that “the diet included rice and beans as the main source of protein”, confirming it was plant based.
- The sex of participants in one study [46] was categorized as “M + F” in the 2014 review [14], but we were able to separately extract data for male and female participants.
- All participants in another study [47] were classified as female in the 2014 review [14], but the original paper included male and female participants. We were able to extract their data separately.
- One study [48] was classified as conducted in a “Tropical” climate based on the 2014 review [14], which referred to researchers from the University of São Paulo (Brazil). However, the original text states that the experiment was conducted during a period with average temperatures of 23.1–24.0 °C. Therefore, the climate classification was revised to “Temperate.”
- In the 2014 review [14], the sex of participants in one study [49] was listed as “male”, but the original text refers only to “medical students of the University of Ibadan.” Since it is unclear whether the university admitted only male students at that time, we classified the sex as “unknown.”
- In one study [16] participants were categorized as “M + F” in the 2014 review [14], but we were able to extract and analyze the male and female variables separately.

**Table 1:** Summary of extracted nitrogen requirements excluding outliers.

Data Source		Climate	Protein	Sex	*n*	Age	Requirement ^2^		
Study ID	Ex ^1^		Source			(y, Median)	Mean	SD	Ref.
01_Clark HE	A	Temperate	Mixed	F	1	24	66.8		[46]
01_Clark HE	B	Temperate	Mixed	M	4	24	92.2	9.9	[46]
02_Alford BB	A	Temperate	Plant-based	F	14	21	123.7	20.9	[50]
03_Cheng AH	A	Temperate	Mixed	M	7	24	148.2	27.3	[51]
03_Cheng AH	B	Temperate	Mixed	M	7	68	138.2	16.3	[51]
04_Uauy R	A	Temperate	Animal-based	F	7	74	145.4	34.1	[47]
04_Uauy R	B	Temperate	Animal-based	M	6	71	102.5	41.6	[47]
05_Thomas MR	A	Temperate	Plant-based	F	7	20	95.4	25.3	[52]
06_Fajardo LF	A	Tropical	Animal-based	F	2	22.5	111.6	2.4	[44]
06_Fajardo LF	B	Tropical	Animal-based	M	4	22.5	133.1	15.2	[44]
06_Fajardo LF	C	Tropical	Plant-based	M	7	23	134.4	16.1	[44]
07_Inoue G	A	Temperate	Animal-based	M	7	21	93.4	9.1	[53]
07_Inoue G	B	Temperate	Plant-based	M	5	22	119.8	15.9	[53]
07_Inoue G	C	Temperate	Mixed	M	8	21	97.7	21.1	[53]
08_Tontisirin K	A	Tropical	Animal-based	M	13	23	138.1	18.8	[54]
09_Bourges H	A	Temperate	Plant-based	M	8	20.5	113.6	20.0	[55]
09_Bourges H	B	Temperate	Animal-based	M	3	21	102.6	19.8	[55]
10_Calloway DH	A	Temperate	Animal-based	F	4	25	85.7	25.3	[56]
11_Huang PC	A	Temperate	Mixed	M	7	24.5	127.4	21.7	[57]
11_Huang PC	B	Temperate	Animal-based	M	5	24.5	95.4	13.1	[57]
12_Yanez E	A	Temperate	Animal-based	M	8	25.5	98.4	5.8	[58]
12_Yanez E	B	Temperate	Mixed	M	7	25	127.0	17.8	[58]
13_Istfan N	A	Temperate	Plant-based	M	8	19.5	94.2	19.2	[59]
14_Scrimshaw NS	A	Temperate	Plant-based	M	13	20	117.7	31.3	[60]
14_Scrimshaw NS	B	Temperate	Animal-based	M	5	19	103.1	9.7	[60]
15_Vannucchi H	A	Tropical	Plant-based	M	8	23	108.9	14.8	[48]
16_Agarwal KN	A	Tropical	Plant-based	F	5	32	104.8	6.9	[61]
16_Agarwal KN	B	Tropical	Plant-based	M	6	32	90.4	10.3	[61]
17_Dutra de Oliveira	A	Tropical	Plant-based	M	8	26.5	115.3	17.2	[45]
18_Hussein MA	A	Tropical	Mixed	F	8	22.5	86.0	6.5	[62]
19_Ozalp I	A	Temperate	Mixed	M	11	23	98.5	15.3	[63]
20-2_Chen XC	A	Temperate	Mixed	M	10	42	147.7	17.9	[21]
21_Young VR	A	Temperate	Plant-based	M	8	19.4	112.2	15.2	[64]
21_Young VR	B	Temperate	Animal-based	M	7	21.1	90.9	45.8	[64]
22_Atinmo T	A	Tropical	Mixed	U ^3^	14	20	119.6	20.4	[49]
23_Kaneko K	A	Temperate	Mixed	F	12	21	103.0	21.4	[65]
24_De Unamuno	A	Tropical	Plant-based	M	7	71	113.1	25.8	[66]
25_Egana JI	A	Tropical	Plant-based	M	7	26	102.8	13.1	[67]
25_Egana JI	B	Tropical	Animal-based	M	5	26	80.1	11.2	[67]
26_Egun GN	A	Tropical	Mixed	F	12	23.5	81.4	2.8	[68]
28_Campbell WW	A	Temperate	Animal-based	M	11	33.5	93.0	32.6	[16]
28_Campbell WW	B	Temperate	Animal-based	F	11	33.5	106.1	18.8	[16]
28_Campbell WW	C	Temperate	Animal-based	M	8	72	104.1	14.7	[16]
28_Campbell WW	D	Temperate	Animal-based	F	10	72	87.6	14.5	[16]
29_Atinmo T	A	Tropical	Mixed	M	18	23	108.6	15.4	[17]
R12_Vargas E	A	Temperate	Plant-based	M	19	28	92.4	11.8	[43]
R12_Vargas E	B	Temperate	Mixed	M	20	26.5	80.2	10.0	[43]
R45_Pasricha S	A	Tropical	Mixed	F	3	26	107.5	6.0	[41]

^1^ Experiment; ^2^ mg/kg/day; ^3^ Unknown.

### 3.3. Nitrogen Requirement

We obtained nitrogen requirement data for 405 individuals (292 males, 98 females, and 15 of unknown sex) from 48 experiments across the 31 literature sources analyzed.

All data were included in the analysis, even from experiments conducted under clearly different conditions. For example, Vargas et al. (Study ID = R12_Vargas) compared two conditions with energy intakes set at 46 and 51 kcal/kg using the same protein source [43]. However, the difference in energy intake was not considered in our analysis.

From the collected data, individuals with extreme nitrogen requirements—defined as less than 50 mg/kg/day (three males, two females) or over 200 mg/kg/day (four males, one unknown)—were excluded. The remaining 395 individuals (285 males, 96 females, 14 unknown) were included in the final analysis (Table 1).

The Supplementary Materials (Supplementary Table S2) provides the nitrogen requirements, along with the slope and intercept of the regression equations used to calculate them, for all 405 participants, including those excluded from further analysis. The metadata used in the proposed method are also included.

### 3.4. Overview of Nitrogen Requirements

The distribution of nitrogen requirements did not follow a normal distribution (*p* < 0.001, Shapiro–Wilk test). However, after natural logarithmic transformation, the distribution became normal (*p* = 0.347). Normality was also observed when data were stratified by sex (male: *p* = 0.539; female: *p* = 0.478; unknown: *p* = 0.603; Shapiro–Wilk test) and by climate (temperate: *p* = 0.415; tropical: *p* = 0.101; Shapiro–Wilk test). However, when classified by protein source, nitrogen requirements from animal protein (*p* = 0.434, Shapiro–Wilk test) and plant protein (*p* = 0.612, Shapiro–Wilk test) followed a normal distribution, while those from mixed protein sources did not (*p* = 0.021, Shapiro–Wilk test). For age categories, nitrogen requirements were normally distributed in both those under 60 years (*p* = 0.355, Shapiro–Wilk test) and those aged 60 years and older (*p* = 0.471, Shapiro–Wilk test). The mean value and 95% CI, calculated from the natural logarithm of nitrogen requirements and back-transformed, are summarized in Table 2.

The mean nitrogen requirement was 104.2 mg N/kg/day overall.

When classified by sex, no significant difference was observed in the natural logarithm of nitrogen requirement between males and females (*p* = 0.58, *t*-test). However, when the 14 individuals classified as “unknown” were reclassified as males, the nitrogen requirement was significantly higher in males (*p* = 0.035).

When classified by climate, no significant difference was observed between the temperate and tropical groups (*p* = 0.176, *t*-test).

When classified by protein source, no significant difference was observed among the animal, plant, and mixed groups (*p* = 0.259, Kruskal–Wallis test).

The mean age of the study participants was 29.9 ± 15.6 years. When classified into two groups based on age (under 60 years: 103.6 mg N/kg/day, 95% CI: 101.1 to 106.2; 60 years and older: 108.9 mg N/kg/day, 95% CI: 99.8 to 118.8), there was no significant difference between the groups (*p* = 0.275).

**Figure 2:**
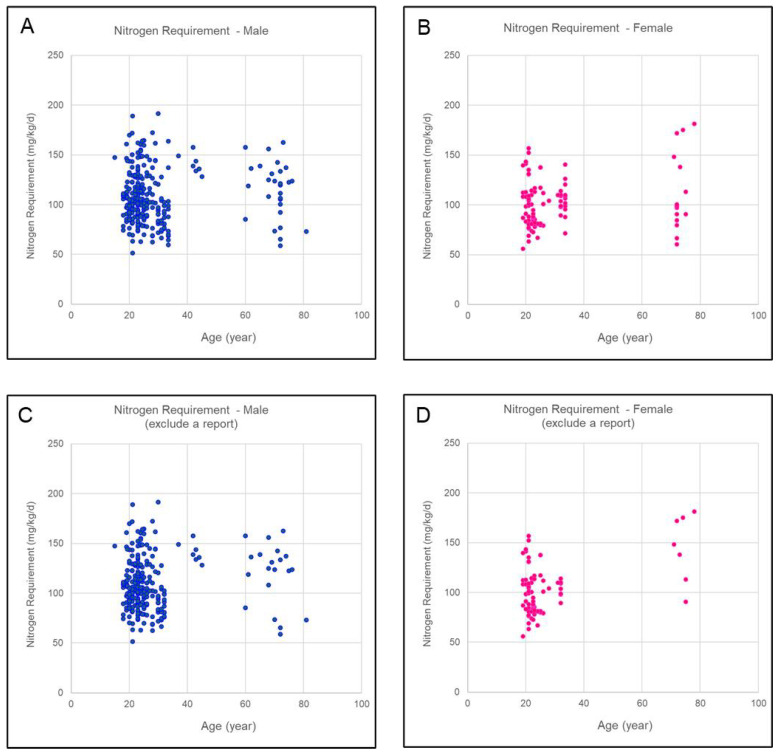
Scatter plots of nitrogen requirements versus age. (**A**) Males (*n* = 285); (**B**) Females (*n* = 96); (**C**) Males, excluding data from reference [16] (*n* = 266); (**D**) Females, excluding data from reference [16] (*n* = 75).

When nitrogen requirements were plotted against age in a scatter plot, the age distribution of participants was skewed toward those under 50 years and those aged 60 years or older. Nitrogen requirements were predominantly within the range of 50–150 mg/kg/day for males and females under 50 years. However, among females aged 60 years or older, a higher proportion of data points exceeded 150 mg/kg/day. This trend became more pronounced when one literature source [16] was excluded from the data distribution (Figure 2).

### 3.5. Meta-Analysis

Of the 395 subjects, 14 with unknown sex [49] and one study with only a single participant (01_Clark HE) [46] were excluded. A meta-analysis was conducted on the remaining 380 participants (285 males and 95 females), with data analyzed separately by sex (Table 3).

The nitrogen requirement for males was 109.1 mg N/kg/day (95% CI: 103.0–115.1). Subgroup analysis based on protein source showed nitrogen requirements of 103.3 mg N/kg/day (95% CI: 94.3–112.4) for animal-based protein and 108.9 mg N/kg/day (95% CI: 100.9–117.0) for plant-based protein. Although the requirement from plant-based protein was higher than that from animal-based protein, the difference was not statistically significant. For mixed protein sources, the nitrogen requirement was 116.0 mg N/kg/day (95% CI: 100.0–132.0).

The nitrogen requirement for females was 102.4 mg N/kg/day (95% CI: 92.3–112.5). Subgroup analysis showed nitrogen requirements of 105.8 mg N/kg/day (95% CI: 91.6–120.0) for animal-based protein and 109.0 mg N/kg/day (95% CI: 94.2–123.8) for plant-based protein. Similar to the male subgroup, the requirement derived from plant-based protein was higher than that from animal-based protein, but the difference was not statistically significant. The nitrogen requirement for mixed protein was 93.7 mg N/kg/day (95% CI: 82.4–105.0).

The heterogeneity of nitrogen requirements across studies was high in all cases, with *I*^2^ = 92% for males and *I*^2^ = 97% for females, indicating substantial heterogeneity. To explore potential sources of this variability, we investigated differences in the protein sources used in the studies. However, the *I*^2^ values remained high across all subgroups, suggesting that differences in protein sources did not significantly contribute to the observed heterogeneity.

The effects of climate on nitrogen requirements were also analyzed (Table 4). Based on the 95% CI, climate did not have a significant impact on nitrogen requirements in either males or females. However, the mean nitrogen requirement for males was higher in tropical regions, whereas the opposite trend was observed for females. Notably, heterogeneity among females in tropical regions was particularly high, with an *I*^2^ value of 99%.

Next, we examined the effect of age on nitrogen requirements using meta-regression analysis stratified by sex. The analysis was performed using both the untransformed nitrogen requirement values (native values) and their log-transformed counterparts. The results showed that age had no significant effect on nitrogen requirements, regardless of sex or whether the values were log-transformed (Table 5).

**Figure 3:**
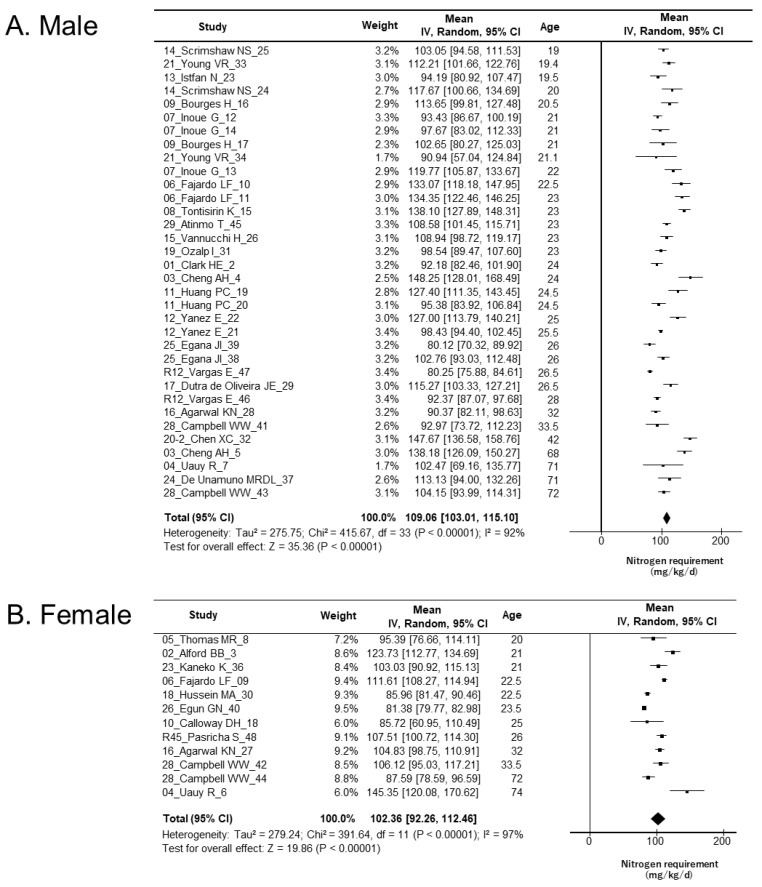
Nitrogen requirements in healthy adults estimated using a random effects model. Studies, which represent the study IDs in Table 1, are arranged by median participant age, from youngest (**top**) to oldest (**bottom**). No clear trend in nitrogen requirement is observed with increasing age. (**A**) Males: Overall nitrogen requirement = 109.06 mg N/kg/day (95% CI: 103.01–115.10). (**B**) Females: Overall nitrogen requirement = 102.36 mg N/kg/day (95% CI: 92.26–112.46).

When nitrogen requirements were plotted in a forest plot according to the median age of each experiment, no apparent relationship between age and nitrogen requirements was observed in either males or females (Figure 3). A similar pattern was observed when the plots were stratified by protein source (animal, plant, or mixed) or climate (temperate or tropical) (Supplementary Figure S1).

## 4. Discussion

### 4.1. Nitrogen Requirements

To date, systematic reviews integrating nitrogen balance test data to estimate protein requirements in human adults have been published in 2003 [5] and 2014 [14]. Rand et al. extracted data from 19 independent nitrogen balance studies involving 235 healthy adults, reporting a median requirement of 104.6 mg N/kg/day (95% CI: 101–110) [5]. Li et al. extracted nitrogen requirements from 28 studies involving 348 individuals, with a mean of 105.6 mg N/kg/day [14]. In this study, nitrogen requirements were extracted from 31 studies involving 405 individuals. After excluding outliers, the nitrogen requirement for 395 individuals was 104.2 mg N/kg/day (95% CI: 101.7–106.7). The representative values obtained in this study are consistent with those reported in the previous two systematic reviews [5,14].

In this study, the evaluation of miscellaneous nitrogen losses (i.e., endogenous losses from sweat, skin, hair, nails, etc.), as well as the adaptation period used for nitrogen balance measurements, were consistent with the methodologies of the two previous systematic reviews [5,14]. Therefore, we conclude that nitrogen requirements can be extracted using the same approach as employed in the earlier reviews [5,14].

### 4.2. Effects of Climate

Rand et al. reported a median nitrogen requirement of 102.8 mg N/kg/day in temperate regions, while the requirement in tropical regions was approximately 10 mg N/kg/day higher (*p* < 0.047) [5]. In contrast, Li et al. reported significantly lower nitrogen requirements in tropical regions (100.48 mg N/kg/day, 95% CI: 96.54–104.59) compared to temperate regions (108.85 mg N/kg/day, 95% CI: 105.64–112.17) [14].

In our analysis, the mean nitrogen requirement calculated from natural logarithms was higher in tropical regions; however, the difference was not statistically significant.

In our dataset, for one study [48], we reclassified the climate as “temperate” instead of “tropical”, as previously reported [14] based on the description in the original article. For other studies, when climate conditions during the experimental period were not explicitly stated, we estimated the climate based on the experimental location or the authors’ affiliations when the location was not specified.

When direct measurements were unavailable, nitrogen requirements were adjusted for miscellaneous losses based on climate classification.

Therefore, variations in climate classification and assumptions regarding miscellaneous nitrogen losses may explain the lack of significant differences in our analysis. The dataset used in this study is available as Supplementary Materials and can be modified and verified as needed.

### 4.3. Effects of Sex

Two previous systematic reviews [5,14] reported that nitrogen requirements were significantly higher in males than in females. However, our analysis revealed no statistically significant difference between males and females.

The two earlier reviews [5,14] classified participants from a 1988 study [49], which involved medical students at the University of Ibadan in Nigeria, as male. However, we found no clear documentation indicating that female medical students were excluded from the university in the 1980s. Since the original text did not explicitly mention the participant’s sex, we classified them as “unknown.” The mean nitrogen requirement of these participants (*n* = 14) was 118.1 mg N/kg/day (95% CI: 107.1–130.1), which was significantly higher than that of males in our dataset. This discrepancy may partly explain the difference from the findings of the previous systematic reviews.

Furthermore, the lack of significant sex-based differences in our dataset may be attributed to the larger sample size (*n* = 395), which exceeds that of Rand et al. (*n* = 235) [5] and Li et al. (*n* = 348) [14].

### 4.4. Effects of Age

Most studies on nitrogen balance have been conducted in participants aged ≤30 or ≥60, resulting in limited data for the intermediate age group. Rand et al. classified participants under 55 as “young” and those over 55 as “old”, but found no significant difference in nitrogen requirements between the two groups [5]. Similarly, Li et al. categorized participants under 60 as “young” and those 60 or older as “old”, and also found no significant differences between the groups [14].

In our analysis, we stratified the dataset by sex and plotted age against nitrogen requirements in scatterplots. Among females aged 60 years and older, nitrogen requirements appeared to be higher. This impression became stronger when data from a particular study paper (28_Campbell) [16] were excluded. However, as there was no clear justification for exclusion, we proceeded with the entire dataset. We divided the data into two groups (≤60 years and >60 years) and compared them; however, we found no significant differences, consistent with previous studies [5,14]. Additionally, meta-regression analyses stratified by sex did not show any significant relationship between age and nitrogen requirement. These findings further support the conclusions of earlier systematic reviews [5,14].

### 4.5. Effect of Protein Source

Rand et al. reported that the source of dietary protein (animal, plant, or mixed) did not significantly affect nitrogen requirements [5]. Similarly, Li et al. reported no effect of protein sources on nitrogen requirements [14]. In our meta-analysis, stratified by sex, we found no significant differences in nitrogen requirements based on protein source for either males or females. Therefore, our findings are consistent with those of the previous studies [5,14].

Rand et al. reported that the absence of a clear difference might be due to the wide availability among plant-based proteins, some of which, such as soy, are comparable in quality to animal proteins, while others, such as wheat protein, have lower utilization efficiency [5]. Notably, the heterogeneity of the included trials exceeded 90% for both males and females. Several studies in our dataset were designed to evaluate the quality or bioavailability of specific protein sources [50,52,59,64,67,69,70], which may have influenced nitrogen requirements. These differences in study aims could contribute to the observed variability and obscure potential effects when categorizing protein sources simply as animal or plant.

### 4.6. Effect of Experimental Protocol

In most nitrogen balance studies, nitrogen intake and the amount of nitrogen excreted in urine and feces are measured. Minor losses through other routes were corrected using estimated values based on previous studies using protein-free diets. However, the participants in nitrogen balance studies are not placed on protein-free diets. Furthermore, if the adaptation period is insufficient, nitrogen balance may not be fully achieved, increasing the risk of underestimating miscellaneous nitrogen losses.

The standard UNU short-term protocol in the early 1980s recommends that participants maintain a constant nitrogen intake for 10–15 consecutive days [71]. In practice, urinary nitrogen excretion typically stabilizes to a statistically nonsignificant level within 10–14 days after switching to a protein-free diet [72]. Therefore, a minimum of 10 days is required per intake level. However, applying this protocol to assess three intake levels in a single participant would require over 30 consecutive days of controlled dietary intake, excretion monitoring, and physical activity regulation, which is challenging in practice. Some studies have reported that nitrogen excretion stabilizes within five [7] or seven days [73] after dietary changes. Notably, even studies conducted after the UNU guidelines were established often did not fully adhere to the standard protocol.

Additionally, the influence of energy intake on protein requirements has been recognized since the time when protein requirements were determined using the factorial method [1]. Energy deficiency leads to an overestimation of nitrogen requirements unless offset by increased physical activity, whereas excessive energy intake can increase nitrogen retention and result in an underestimation of nitrogen requirements [71]. Therefore, in nitrogen balance studies, physical activity was often restricted, and energy intake was adjusted to estimated average requirements. However, the standard deviation of estimated energy requirements for adult males is approximately 200 kcal [74], and basal metabolic rate declines with age after 18 years, varying considerably even within the same age group [75]. Nevertheless, most nitrogen balance studies did not base energy intake on directly measured energy expenditure in individual participants. This likely contributes to variability in observed nitrogen requirements across studies.

Therefore, the lack of clear and consistent findings regarding the effect of climate, sex, age, and protein source on nitrogen requirements in previous reports [5,14] as well as in the current meta-analysis, may be attributable to methodological limitations and variability in nitrogen balance study protocols. In addition, methodological variation could also account for the high heterogeneity.

### 4.7. Application of the Nitrogen Requirements Based on the Nitrogen Balance Method to Reference Values

The nitrogen requirements calculated in this study were consistent with those of previous meta-analyses [5,14]. These meta-analyses have been used to determine protein reference values for adults, including the 2017 revision of protein recommendations by D-A-CH [10] and Dietary Reference Intakes (DRIs) for Japanese (2025) [76]. Specifically, the protein reference value is calculated by multiplying 105 mg N/kg/day by 6.25, assuming 16% of protein is nitrogen.

However, the nitrogen requirement determined by the nitrogen balance method represents the minimum amount necessary to maintain nitrogen levels in the body without losing weight. An adaptation period of at least five days is necessary to calculate this value. Therefore, for healthy people with a normal physical activity level of 1.75 times their basal metabolic rate, the nitrogen requirement based on the nitrogen balance method should be smaller than the daily nitrogen turnover rate. Thus, a different standard should be used to determine the protein recommendation for healthy, active individuals.

Considering this, the latest Japanese DRIs (2025) [76] use nitrogen requirements based on the nitrogen balance method to set the estimated average requirement and avoid deficiency. Meanwhile, they set the dietary goal based on the energy ratio of protein. For example, for men aged 18–29 with a reference weight of 63 kg, the estimated average requirement and dietary goal are 50 g/day and 13–20% EN, respectively. Accordingly, the daily protein goal for individuals with a high physical activity level of 2.0 and an estimated energy requirement of 3000 kcal/day is 97.5–150 g (1.5–2.4 g/kg), which is almost equivalent to the recommended intake for athletes (1.4–2.4 g/kg) [77,78,79,80,81].

Therefore, to avoid deficiency, the nitrogen requirement based on the nitrogen balance method should be used. However, different criteria should be used to establish reference values for healthy living. In addition to the energy ratios used by Japanese DRIs (2025), the IAAO method is also a candidate, though the scientific evidence supporting it is limited [13]. Further research is warranted.

### 4.8. Usefulness and Limitations of the Dataset

This study’s nitrogen requirements dataset includes more individual studies than the two previously published systematic reviews [5,14]. As the meta-analysis shows, the *I*^2^ value is very high, indicating substantial heterogeneity in the reported nitrogen requirements across individual studies. Therefore, the integrated values should not be considered highly reliable. However, because results from studies conducted under various conditions were compiled, this dataset is expected to provide a rough indication of the minimum nitrogen requirement level for adult humans.

The nitrogen requirement for males should be higher than that for females when expressed per kilogram of body weight. This is because males generally have a lower body fat percentage and a higher lean body mass percentage. In addition, the age-related decrease in basal metabolic rate [75] should also influence nitrogen requirements. However, this study was unable to confirm these expected trends, which may be due to the analysis of a highly heterogeneous dataset. Future research may provide clearer answers by reviewing the experimental conditions of the studies included in this dataset and extracting relevant data accordingly.

Of the 75 papers identified on PubMed that may contain data on individual nitrogen balance or requirements based on their titles and abstracts, we were unable to obtain 17 papers (Supplementary Table S1). The dataset provided by this research (Supplementary Table S2) can be made more comprehensive by retrieving and incorporating data from these papers.

The primary objective of this study was to provide a comprehensive dataset that includes data used in the previous meta-analyses [5,14], so the selection of literature and data extraction were conducted following the same procedure. As a result, a very high heterogeneity (*I*^2^ > 90%) was observed. This suggests that variables other than age, gender, and climate may be involved. We did not assess the risk of bias in the included literature using methods such as ROBINS-I. Additionally, we did not extract individual energy intake, although energy intake has been well known to influence nitrogen balance [1]. These may contribute the high heterogeneity. However, restricting data based on bias risk or the lack of reporting of individual energy intake would not align with the objective of this study. Meanwhile, since the comprehensive dataset is publicly provided (Supplementary Table S2), researchers can extract data based on various considerations, such as bias risk and energy intake, and use more advanced statistical methods (e.g., multistage analysis) to conduct a more comprehensive analysis from multiple perspectives. That is what this research aims to achieve. The dataset was compiled with the expectation that it would serve as a foundation for future research.

For this study, we incorporated the literature from the previous two meta-analyses [5,14] and conducted an additional systematic literature search to compile all relevant studies. We used the same method as in the previous two papers [5,14] for extracting data and calculating nitrogen requirements. Meta-analyses of the compiled dataset confirmed that the outcomes were almost consistent with those of the previous two papers [5,14]. These procedures were conducted in accordance with the PRISMA guidelines, except as noted below. As this is the third systematic review on the nitrogen requirements based on nitrogen balance, and since the main objective was to compile a comprehensive dataset, the review was not registered with PROSPERO. The risk of bias assessment and sensitivity analysis were not performed. This is because the study included outdated literature, and the goal was to compile as many references as possible. In addition, GRADE assessment of the meta-analysis outcomes was not performed because evaluating the quality of the evidence of the outcomes was not the main objective of this study. Therefore, in a strict sense, some may consider this study not to comply with the definition of a systematic review.

### 4.9. Future Prospects

Nitrogen requirements based on nitrogen balance have been used to determine protein requirements. However, conducting new studies is difficult from both ethical and practical standpoints. Therefore, this study compiled a comprehensive dataset (Supplementary Table S2) of individual nitrogen requirements reported in previously published papers. We hope that the proposed dataset will serve as a foundation for reevaluating protein recommendations and developing strategies to combat malnutrition.

Nitrogen balance measurements were performed on subjects who had adapted to a certain protein level. Therefore, they do not necessarily reflect the requirements of free-living individuals. Based on these studies and previous factorial method studies, Millward concluded that when considering human nitrogen balance, it is necessary to consider not only nitrogen but also individual amino acids in terms of obligatory and adaptive metabolic demands [82]. In the same paper, Millward elucidates and explains the currently widely used essential amino acid scoring patterns and issues surrounding essential amino acids [82]. Furthermore, since humans can synthesize lysine using urea nitrogen [83], previous amino acid-based studies may need to be reexamined. Millward also summarized the methodological considerations for determining protein and amino acid requirements using nitrogen balance and IAAO methods [9]. A review of our dataset in light of these summarized issues and new findings is expected to provide fresh insights into nitrogen metabolism.

## 5. Conclusions

This systematic review collected and provided a comprehensive dataset on adult nitrogen requirements obtained using the nitrogen balance method over the past half-century. Meta-analyses confirmed the consistency with previous studies. The compiled dataset is provided as a supplementary table. Because the nitrogen balance method requires intensive intervention of study participants, it is becoming increasingly difficult to implement due to ethical and practical concerns. However, the nitrogen requirements based on the nitrogen balance method should continue to be used to avoid protein deficiencies. Physiologically, this method reflects human adaptation to low protein levels, though the underlying mechanism remains unclear. Therefore, the individual-level dataset organized in this study is expected to serve as an essential foundation for reevaluating nitrogen requirements based on nitrogen balance studies.

## Figures and Tables

**Table 2:** Summary of nitrogen requirements included in this review.

		*n*	Nitrogen Requirement (mg/kg/Day)			
			Mean ^1^	95% CI ^1^		
Sex	Male	285	105.2	102.2	–	108.2
	Female	96	99.6	94.9	–	104.5
	Unknown	14	118.1	107.1	–	130.2
Climate	Temperate	276	103.1	100.0	–	106.2
	Tropical	119	106.9	102.8	–	111.1
Protein source	Animal	116	101.4	96.4	–	106.6
	Plant	130	106.7	103.1	–	110.5
	Mixed	149	104.3	100.2	–	108.5
Total		395	104.2	101.7	–	106.7

^1^ Calculated from natural logarithm of nitrogen requirement.

**Table 3:** Meta-analysis of nitrogen requirement in healthy adults (random effects model).

Sex	Protein Source	Study No.	Nitrogen Requirement (mg N/kg/day)	95% CI	*I* ^2^
Male	All	34	109.1	103.0–115.1	92%
	Animal-based	12	103.3	94.3–112.4	88%
	Plant-based	12	108.9	100.9–117.0	85%
	Mixed	10	116.0	100.0–132.0	96%
Female	All	12	102.4	92.3–112.5	97%
	Animal-based	5	105.8	91.6–120.0	89%
	Plant-based	3	109.0	94.2–123.8	81%
	Mixed	4	93.7	82.4–105.0	95%

Two studies, 01_Clark HE (Ex. A) and 22_Atinmo T (Ex. A), from Table 1 were excluded from the meta-analysis.

**Table 4:** Effects of climate on nitrogen requirements in healthy adults (random effects model).

Sex	Climate	Study No.	Nitrogen Requirement (mg N/kg/Day)	95% CI	*I* ^2^
Male	Temperate	25	107.7	101.0–114.5	91%
	Tropical	9	112.5	99.3–125.7	93%
Female	Temperate	7	106.0	92.7–119.3	84%
	Tropical	5	98.2	83.4–113.0	99%

Two studies, 01_Clark HE (Ex. A) and 22_Atinmo T (Ex. A), from Table 1 were excluded from the meta-analysis.

**Table 5:** Effect of age on nitrogen requirements in healthy adults analyzed by meta-regression analysis (random effects model).

Sex	Nitrogen Requirement (mg N/kg/Day)	Estimate	SE	*p*
Male	Untransformed values	0.1815	0.2161	0.407
	Log transformed values	0.001394	0.001974	0.485
Female	Untransformed values	0.2255	0.2867	0.450
	Log transformed values	0.001559	0.00271	0.578

Two studies, 01_Clark HE (Ex. A) and 22_Atinmo T (Ex. A), from Table 1 were excluded from the meta-regression analysis.

## Data Availability

The original contributions presented in this study are included in the article/Supplementary Materials. Further inquiries can be directed to the corresponding author.

## Supplementary Materials

The following supporting information can be downloaded at: https://www.mdpi.com/article/10.3390/nu17162615/s1, Figure S1: Forest plots; Table S1: List of unobtained papers; Table S2: Comprehensive dataset of nitrogen requirements and metadata.

## Abbreviations

The following abbreviations were used in this manuscript:

CONSORT	Consolidated Standards of Reporting Trials
PRISMA	Preferred Reporting Items for Systematic Reviews and Meta-analyses
GRADE	Grading Recommendations Assessment, Development, and Evaluation
