# Supplementary material for: Nitrogen Requirements in Healthy Adults: A Systematic Review and Meta-Analysis of Nitrogen Balance Studies
Source: Nutrients. 2025 Aug 12;17(16):2615. doi: 10.3390/nu17162615 (PMC12389376; doi:10.3390/nu17162615)
Supplement: Supplementary file 1 [file nutrients-17-02615-s001.zip › Table_S1.pdf]

Table S1 List of the literatures un-obtained

#	First author	Year	PMID	Authors	Journal. year months, vol(issue): pages	Ref
1	CONSOLAZIO CF	1962	14022658	CONSOLAZIO CF, NELSON RA, MATOUSH LO, HARDING RS, CANHAM JE.	Rep US Army Med Res Nutr Lab Denver. 1962 Oct 1:22p.	24
2	Drozdova TM	1979	462890	Drozdova TM.	Vopr Pitan. 1979 May-Jun;(3):32-5.	25
3	Gonţea I	1965	5843030	Gonţea I, Suţescu P, Dumitrache S, Cocora D, Suma S.	Fiziol Norm Patol. 1965 Mar-Apr;11(2):133-42.	26
4	Gontzea I	1968	5681863	Gontzea I, Sutzesco P, Dumitrache S.	Ann Nutr Aliment. 1968;22(4):183-236.	27
5	GONTZEA I	1962	13949007	GONTZEA I, SUTZESCO P, DUMITRACHE S.	Arch Sci Physiol (Paris). 1962;16:127-38.	28
6	GONTZEA I	1959	13850968	GONTZEA I, SUTZESCOU P, DUMITRACHE S.	Arch Sci Physiol (Paris). 1959;13:99-108.	29
7	GONTZEA J	1960	13706892	GONTZEA J, SUTESCU P, DIMITRAKIS S.	Vopr Pitan. 1960 Nov-Dec;19:12-7.	30
8	Grigorov IuG	1972	5046696	Grigorov IuG, Solomko GI.	Vrach Delo. 1972 Mar;3:35-7.	31
9	Hartig W	1971	5005793	Hartig W, Czarnetzki HD, Keitel R.	Z Exp Chir. 1971;4(2):121-8.	32
10	Iatsyshina TA	1982	6891144	Iatsyshina TA, Brents MIa, Mamaeva EM.	Vopr Pitan. 1982 Sep-Oct;(5):27-33.	33
11	Iatsyshina TA	1983	6684363	Iatsyshina TA, Vysotskiĭ VG, Safronova AM, Eganian RA.	Vopr Pitan. 1983 May-Jun;(3):22-4.	34
12	Lin T	1973	4805335	Lin T, Chen ML, Chen JS.	Chin J Physiol. 1973 Dec 31;21(3):143-50.	35
13	SOENKE ML	1947	20240837	SOENKE ML, HORNING MG, WATSON EH.	Am Pract Dig Treat. 1947 May;1(9):489-92.	36
14	SOENKE ML	1947	20286867	SOENKE ML, HORNING MG, WATSON EH.	Am Pract Dig Treat. 1947 Jan;1(5):276-82.	37
15	Vankhanen VD	1969	5368109	Vankhanen VD, Chistiakova AM.	Vopr Pitan. 1969 Jul-Aug;28(4):33-9.	38
16	Vysotskiĭ VG	1977	883212	Vysotskiĭ VG, Kochetkova AN, Iatsyshina TA.	Vopr Pitan. 1977 Jan-Feb;(1):3-9.	39
17	Vysotskiĭ VG	1978	726379	Vysotskiĭ VG.	Vopr Pitan. 1978 Nov-Dec;(6):8-17.	40
