# Supplementary material for: Nitrogen Requirements in Healthy Adults: A Systematic Review and Meta-Analysis of Nitrogen Balance Studies
Source: Nutrients. 2025 Aug 12;17(16):2615. doi: 10.3390/nu17162615 (PMC12389376; doi:10.3390/nu17162615)

Supplementary Figure S1

A. Male Animal Protein
B. Male Plant Protein
C. Male Mixed Protein
D. Female Animal Protein
E. Female Plant Protein
F. Female Mixed Protein
G. Male Temperate
H. Male Tropical
I. Female Temperate
J. Female Tropical

# Supplement Figure S1. A

## Male Animal Protein

| Study | Weight | Mean IV, Random, 95% CI | Age |
|---|---|---|---|
| 14_Scrimshaw NS_25 | 10.0% | 103.05 [94.58, 111.53] | 19 |
| 21_Young VR_33 | 0.0% | 112.21 [101.66, 122.76] | 19.4 |
| 13_Istfan N_23 | 0.0% | 94.19 [80.92, 107.47] | 19.5 |
| 14_Scrimshaw NS_24 | 0.0% | 117.67 [100.66, 134.69] | 20 |
| 09_Bourges H_16 | 0.0% | 113.65 [99.81, 127.48] | 20.5 |
| 07_Inoue G_12 | 10.3% | 93.43 [86.67, 100.19] | 21 |
| 07_Inoue G_14 | 0.0% | 97.67 [83.02, 112.33] | 21 |
| 09_Bourges H_17 | 6.5% | 102.65 [80.27, 125.03] | 21 |
| 21_Young VR_34 | 4.3% | 90.94 [57.04, 124.84] | 21.1 |
| 07_Inoue G_13 | 0.0% | 119.77 [105.87, 133.67] | 22 |
| 06_Fajardo LF_10 | 8.4% | 133.07 [118.18, 147.95] | 22.5 |
| 06_Fajardo LF_11 | 0.0% | 134.35 [122.46, 146.25] | 23 |
| 08_Tontisirin K_15 | 9.6% | 138.10 [127.89, 148.31] | 23 |
| 29_Atinmo T_45 | 0.0% | 108.58 [101.45, 115.71] | 23 |
| 15_Vannucchi H_26 | 0.0% | 108.94 [98.72, 119.17] | 23 |
| 19_Ozalp I_31 | 0.0% | 98.54 [89.47, 107.60] | 23 |
| 01_Clark HE_2 | 0.0% | 92.18 [82.46, 101.90] | 24 |
| 03_Cheng AH_4 | 0.0% | 148.25 [128.01, 168.49] | 24 |
| 11_Huang PC_19 | 0.0% | 127.40 [111.35, 143.45] | 24.5 |
| 11_Huang PC_20 | 9.3% | 95.38 [83.92, 106.84] | 24.5 |
| 12_Yanez E_22 | 0.0% | 127.00 [113.79, 140.21] | 25 |
| 12_Yanez E_21 | 10.7% | 98.43 [94.40, 102.45] | 25.5 |
| 25_Egana JI_39 | 9.7% | 80.12 [70.32, 89.92] | 26 |
| 25_Egana JI_38 | 0.0% | 102.76 [93.03, 112.48] | 26 |
| R12_Vargas E_47 | 0.0% | 80.25 [75.88, 84.61] | 26.5 |
| 17_Dutra de Oliveira JE_29 | 0.0% | 115.27 [103.33, 127.21] | 26.5 |
| R12_Vargas E_46 | 0.0% | 92.37 [87.07, 97.68] | 28 |
| 16_Agarwal KN_28 | 0.0% | 90.37 [82.11, 98.63] | 32 |
| 28_Campbell WW_41 | 7.3% | 92.97 [73.72, 112.23] | 33.5 |
| 20-2_Chen XC_32 | 0.0% | 147.67 [136.58, 158.76] | 42 |
| 03_Cheng AH_5 | 0.0% | 138.18 [126.09, 150.27] | 68 |
| 04_Uauy R_7 | 4.4% | 102.47 [69.16, 135.77] | 71 |
| 24_De Unamuno MRDL_37 | 0.0% | 113.13 [94.00, 132.26] | 71 |
| 28_Campbell WW_43 | 9.6% | 104.15 [93.99, 114.31] | 72 |
| **Total (95% CI)** | **100.0%** | **103.32 [94.29, 112.35]** | |

Heterogeneity: Tau² = 194.24; Chi² = 94.88, df = 11 (P < 0.00001); I² = 88%
Test for overall effect: Z = 22.44 (P < 0.00001)

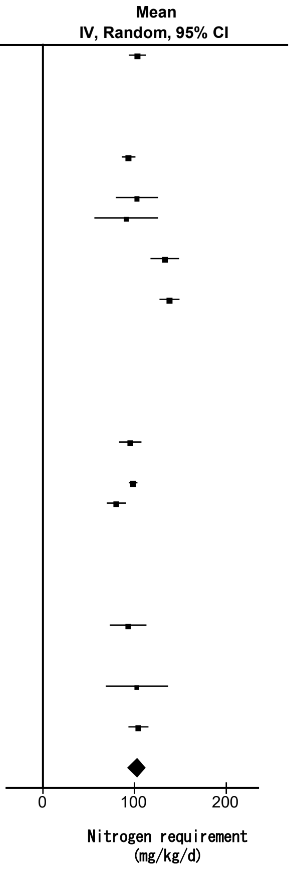

# Supplement Figure S1. B

## Male Plant Protein

| Study | Weight | Mean IV, Random, 95% CI | Age |
|---|---|---|---|
| 14_Scrimshaw NS_25 | 0.0% | 103.05 [94.58, 111.53] | 19 |
| 21_Young VR_33 | 8.8% | 112.21 [101.66, 122.76] | 19.4 |
| 13_Istfan N_23 | 8.1% | 94.19 [80.92, 107.47] | 19.5 |
| 14_Scrimshaw NS_24 | 7.1% | 117.67 [100.66, 134.69] | 20 |
| 09_Bourges H_16 | 7.9% | 113.65 [99.81, 127.48] | 20.5 |
| 07_Inoue G_12 | 0.0% | 93.43 [86.67, 100.19] | 21 |
| 07_Inoue G_14 | 0.0% | 97.67 [83.02, 112.33] | 21 |
| 09_Bourges H_17 | 0.0% | 102.65 [80.27, 125.03] | 21 |
| 21_Young VR_34 | 0.0% | 90.94 [57.04, 124.84] | 21.1 |
| 07_Inoue G_13 | 7.9% | 119.77 [105.87, 133.67] | 22 |
| 06_Fajardo LF_10 | 0.0% | 133.07 [118.18, 147.95] | 22.5 |
| 06_Fajardo LF_11 | 8.4% | 134.35 [122.46, 146.25] | 23 |
| 08_Tontisirin K_15 | 0.0% | 138.10 [127.89, 148.31] | 23 |
| 29_Atinmo T_45 | 0.0% | 108.58 [101.45, 115.71] | 23 |
| 15_Vannucchi H_26 | 8.8% | 108.94 [98.72, 119.17] | 23 |
| 19_Ozalp I_31 | 0.0% | 98.54 [89.47, 107.60] | 23 |
| 01_Clark HE_2 | 0.0% | 92.18 [82.46, 101.90] | 24 |
| 03_Cheng AH_4 | 0.0% | 148.25 [128.01, 168.49] | 24 |
| 11_Huang PC_19 | 0.0% | 127.40 [111.35, 143.45] | 24.5 |
| 11_Huang PC_20 | 0.0% | 95.38 [83.92, 106.84] | 24.5 |
| 12_Yanez E_22 | 0.0% | 127.00 [113.79, 140.21] | 25 |
| 12_Yanez E_21 | 0.0% | 98.43 [94.40, 102.45] | 25.5 |
| 25_Egana JI_39 | 0.0% | 80.12 [70.32, 89.92] | 26 |
| 25_Egana JI_38 | 9.0% | 102.76 [93.03, 112.48] | 26 |
| R12_Vargas E_47 | 0.0% | 80.25 [75.88, 84.61] | 26.5 |
| 17_Dutra de Oliveira JE_29 | 8.4% | 115.27 [103.33, 127.21] | 26.5 |
| R12_Vargas E_46 | 9.9% | 92.37 [87.07, 97.68] | 28 |
| 16_Agarwal KN_28 | 9.3% | 90.37 [82.11, 98.63] | 32 |
| 28_Campbell WW_41 | 0.0% | 92.97 [73.72, 112.23] | 33.5 |
| 20-2_Chen XC_32 | 0.0% | 147.67 [136.58, 158.76] | 42 |
| 03_Cheng AH_5 | 0.0% | 138.18 [126.09, 150.27] | 68 |
| 04_Uauy R_7 | 0.0% | 102.47 [69.16, 135.77] | 71 |
| 24_De Unamuno MRDL_37 | 6.5% | 113.13 [94.00, 132.26] | 71 |
| 28_Campbell WW_43 | 0.0% | 104.15 [93.99, 114.31] | 72 |
| **Total (95% CI)** | **100.0%** | **108.91 [100.87, 116.95]** | |

Heterogeneity: Tau² = 162.98; Chi² = 72.77, df = 11 (P < 0.00001); I² = 85%
Test for overall effect: Z = 26.55 (P < 0.00001)

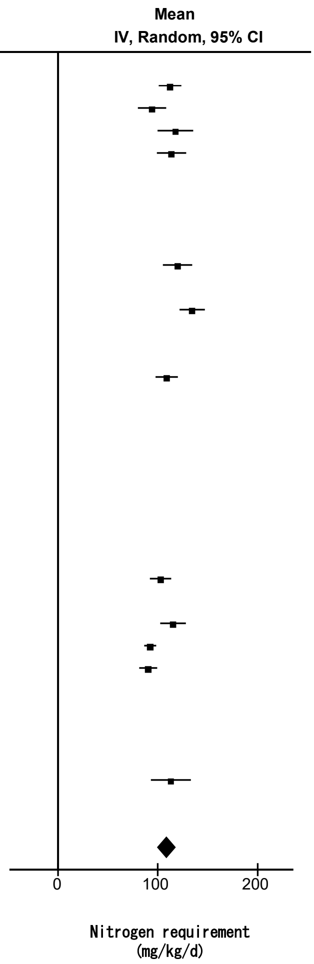

# Supplement Figure S1. C

## Male Mixed Protein

| Study | Weight | Mean IV, Random, 95% CI | Age |
|---|---|---|---|
| 14_Scrimshaw NS_25 | 0.0% | 103.05 [94.58, 111.53] | 19 |
| 21_Young VR_33 | 0.0% | 112.21 [101.66, 122.76] | 19.4 |
| 13_Istfan N_23 | 0.0% | 94.19 [80.92, 107.47] | 19.5 |
| 14_Scrimshaw NS_24 | 0.0% | 117.67 [100.66, 134.69] | 20 |
| 09_Bourges H_16 | 0.0% | 113.65 [99.81, 127.48] | 20.5 |
| 07_Inoue G_12 | 0.0% | 93.43 [86.67, 100.19] | 21 |
| 07_Inoue G_14 | 9.8% | 97.67 [83.02, 112.33] | 21 |
| 09_Bourges H_17 | 0.0% | 102.65 [80.27, 125.03] | 21 |
| 21_Young VR_34 | 0.0% | 90.94 [57.04, 124.84] | 21.1 |
| 07_Inoue G_13 | 0.0% | 119.77 [105.87, 133.67] | 22 |
| 06_Fajardo LF_10 | 0.0% | 133.07 [118.18, 147.95] | 22.5 |
| 06_Fajardo LF_11 | 0.0% | 134.35 [122.46, 146.25] | 23 |
| 08_Tontisirin K_15 | 0.0% | 138.10 [127.89, 148.31] | 23 |
| 29_Atinmo T_45 | 10.4% | 108.58 [101.45, 115.71] | 23 |
| 15_Vannucchi H_26 | 0.0% | 108.94 [98.72, 119.17] | 23 |
| 19_Ozalp I_31 | 10.3% | 98.54 [89.47, 107.60] | 23 |
| 01_Clark HE_2 | 10.2% | 92.18 [82.46, 101.90] | 24 |
| 03_Cheng AH_4 | 9.1% | 148.25 [128.01, 168.49] | 24 |
| 11_Huang PC_19 | 9.6% | 127.40 [111.35, 143.45] | 24.5 |
| 11_Huang PC_20 | 0.0% | 95.38 [83.92, 106.84] | 24.5 |
| 12_Yanez E_22 | 9.9% | 127.00 [113.79, 140.21] | 25 |
| 12_Yanez E_21 | 0.0% | 98.43 [94.40, 102.45] | 25.5 |
| 25_Egana JI_39 | 0.0% | 80.12 [70.32, 89.92] | 26 |
| 25_Egana JI_38 | 0.0% | 102.76 [93.03, 112.48] | 26 |
| R12_Vargas E_47 | 10.6% | 80.25 [75.88, 84.61] | 26.5 |
| 17_Dutra de Oliveira JE_29 | 0.0% | 115.27 [103.33, 127.21] | 26.5 |
| R12_Vargas E_46 | 0.0% | 92.37 [87.07, 97.68] | 28 |
| 16_Agarwal KN_28 | 0.0% | 90.37 [82.11, 98.63] | 32 |
| 28_Campbell WW_41 | 0.0% | 92.97 [73.72, 112.23] | 33.5 |
| 20-2_Chen XC_32 | 10.1% | 147.67 [136.58, 158.76] | 42 |
| 03_Cheng AH_5 | 10.0% | 138.18 [126.09, 150.27] | 68 |
| 04_Uauy R_7 | 0.0% | 102.47 [69.16, 135.77] | 71 |
| 24_De Unamuno MRDL_37 | 0.0% | 113.13 [94.00, 132.26] | 71 |
| 28_Campbell WW_43 | 0.0% | 104.15 [93.99, 114.31] | 72 |
| **Total (95% CI)** | **100.0%** | **115.98 [99.98, 131.97]** | |

Heterogeneity: Tau² = 626.04; Chi² = 245.32, df = 9 (P < 0.00001); I² = 96%
Test for overall effect: Z = 14.21 (P < 0.00001)

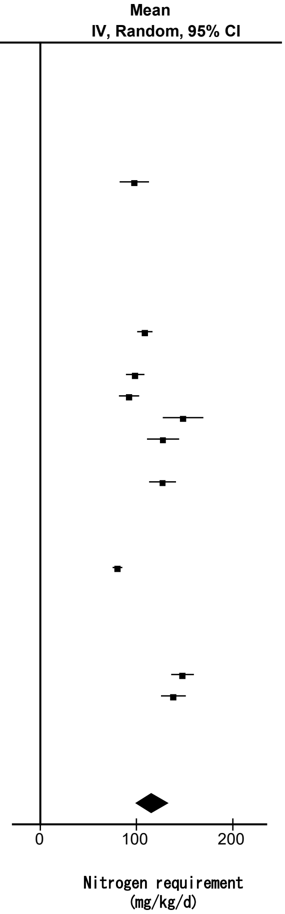

# Supplement Figure S1. D

## Female Animal Protein

| Study | Weight | Mean IV, Random, 95% CI | Age |
|---|---|---|---|
| 05_Thomas MR_8 | 0.0% | 95.39 [76.66, 114.11] | 20 |
| 02_Alford BB_3 | 0.0% | 123.73 [112.77, 134.69] | 21 |
| 23_Kaneko K_36 | 0.0% | 103.03 [90.92, 115.13] | 21 |
| 06_Fajardo LF_09 | 25.5% | 111.61 [108.27, 114.94] | 22.5 |
| 18_Hussein MA_30 | 0.0% | 85.96 [81.47, 90.46] | 22.5 |
| 26_Egun GN_40 | 0.0% | 81.38 [79.77, 82.98] | 23.5 |
| 10_Calloway DH_18 | 14.5% | 85.72 [60.95, 110.49] | 25 |
| R45_Pasricha S_48 | 0.0% | 107.51 [100.72, 114.30] | 26 |
| 16_Agarwal KN_27 | 0.0% | 104.83 [98.75, 110.91] | 32 |
| 28_Campbell WW_42 | 22.3% | 106.12 [95.03, 117.21] | 33.5 |
| 28_Campbell WW_44 | 23.4% | 87.59 [78.59, 96.59] | 72 |
| 04_Uauy R_6 | 14.2% | 145.35 [120.08, 170.62] | 74 |
| **Total (95% CI)** | **100.0%** | **105.81 [91.58, 120.03]** | |

Heterogeneity: $Tau^2 = 203.65$; $Chi^2 = 35.63$, $df = 4$ ($P < 0.00001$); $I^2 = 89\%$
Test for overall effect: $Z = 14.58$ ($P < 0.00001$)

**Mean IV, Random, 95% CI**

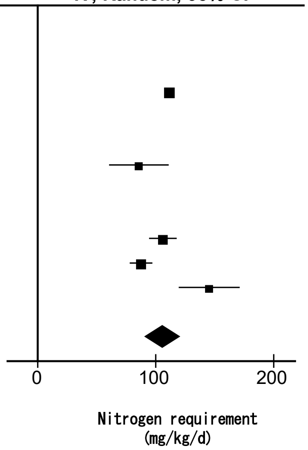

# Supplement Figure S1. E

## Female Plant Protein

| Study | Weight | Mean IV, Random, 95% CI | Age | Mean IV, Random, 95% CI |
|---|---|---|---|---|
| 05_Thomas MR_8 | 25.4% | 95.39 [76.66, 114.11] | 20 | |
| 02_Alford BB_3 | 34.7% | 123.73 [112.77, 134.69] | 21 | |
| 23_Kaneko K_36 | 0.0% | 103.03 [90.92, 115.13] | 21 | |
| 06_Fajardo LF_09 | 0.0% | 111.61 [108.27, 114.94] | 22.5 | |
| 18_Hussein MA_30 | 0.0% | 85.96 [81.47, 90.46] | 22.5 | |
| 26_Egun GN_40 | 0.0% | 81.38 [79.77, 82.98] | 23.5 | |
| 10_Calloway DH_18 | 0.0% | 85.72 [60.95, 110.49] | 25 | |
| R45_Pasricha S_48 | 0.0% | 107.51 [100.72, 114.30] | 26 | |
| 16_Agarwal KN_27 | 40.0% | 104.83 [98.75, 110.91] | 32 | |
| 28_Campbell WW_42 | 0.0% | 106.12 [95.03, 117.21] | 33.5 | |
| 28_Campbell WW_44 | 0.0% | 87.59 [78.59, 96.59] | 72 | |
| 04_Uauy R_6 | 0.0% | 145.35 [120.08, 170.62] | 74 | |
| **Total (95% CI)** | **100.0%** | **108.99 [94.21, 123.76]** | | |

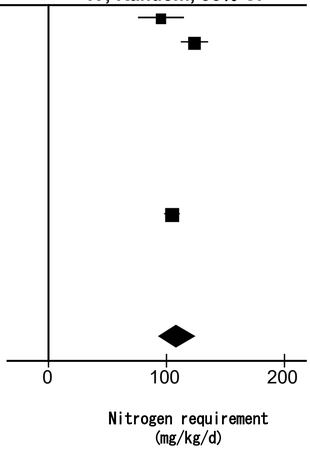


Heterogeneity: Tau² = 132.55; Chi² = 10.69, df = 2 (P = 0.005); I² = 81%
Test for overall effect: Z = 14.46 (P < 0.00001)

# Supplement Figure S1. F

## Female Mixed Protein

| Study | Weight | Mean IV, Random, 95% CI | Age |
|---|---|---|---|
| 05_Thomas MR_8 | 0.0% | 95.39 [76.66, 114.11] | 20 |
| 02_Alford BB_3 | 0.0% | 123.73 [112.77, 134.69] | 21 |
| 23_Kaneko K_36 | 21.0% | 103.03 [90.92, 115.13] | 21 |
| 06_Fajardo LF_09 | 0.0% | 111.61 [108.27, 114.94] | 22.5 |
| 18_Hussein MA_30 | 26.5% | 85.96 [81.47, 90.46] | 22.5 |
| 26_Egun GN_40 | 27.5% | 81.38 [79.77, 82.98] | 23.5 |
| 10_Calloway DH_18 | 0.0% | 85.72 [60.95, 110.49] | 25 |
| R45_Pasricha S_48 | 25.1% | 107.51 [100.72, 114.30] | 26 |
| 16_Agarwal KN_27 | 0.0% | 104.83 [98.75, 110.91] | 32 |
| 28_Campbell WW_42 | 0.0% | 106.12 [95.03, 117.21] | 33.5 |
| 28_Campbell WW_44 | 0.0% | 87.59 [78.59, 96.59] | 72 |
| 04_Uauy R_6 | 0.0% | 145.35 [120.08, 170.62] | 74 |
| **Total (95% CI)** | **100.0%** | **93.69 [82.41, 104.97]** | |

Heterogeneity: Tau² = 119.90; Chi² = 65.90, df = 3 (P < 0.00001); I² = 95%
Test for overall effect: Z = 16.28 (P < 0.00001)

Mean IV, Random, 95% CI

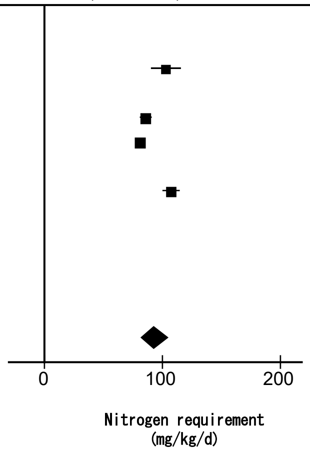

# Supplement Figure S1. G

## Male Temperate

| Study | Weight | Mean IV, Random, 95% CI | Age |
|---|---|---|---|
| 14_Scrimshaw NS_25 | 4.5% | 103.05 [94.58, 111.53] | 19 |
| 21_Young VR_33 | 4.3% | 112.21 [101.66, 122.76] | 19.4 |
| 13_Istfan N_23 | 4.1% | 94.19 [80.92, 107.47] | 19.5 |
| 14_Scrimshaw NS_24 | 3.7% | 117.67 [100.66, 134.69] | 20 |
| 09_Bourges H_16 | 4.0% | 113.65 [99.81, 127.48] | 20.5 |
| 07_Inoue G_12 | 4.6% | 93.43 [86.67, 100.19] | 21 |
| 07_Inoue G_14 | 3.9% | 97.67 [83.02, 112.33] | 21 |
| 09_Bourges H_17 | 3.2% | 102.65 [80.27, 125.03] | 21 |
| 21_Young VR_34 | 2.2% | 90.94 [57.04, 124.84] | 21.1 |
| 07_Inoue G_13 | 4.0% | 119.77 [105.87, 133.67] | 22 |
| 06_Fajardo LF_10 | | Not estimable | 22.5 |
| 06_Fajardo LF_11 | | Not estimable | 23 |
| 08_Tontisirin K_15 | | Not estimable | 23 |
| 29_Atinmo T_45 | 0.0% | 108.58 [101.45, 115.71] | 23 |
| 15_Vannucchi H_26 | 4.4% | 108.94 [98.72, 119.17] | 23 |
| 19_Ozalp I_31 | 4.5% | 98.54 [89.47, 107.60] | 23 |
| 01_Clark HE_2 | 4.4% | 92.18 [82.46, 101.90] | 24 |
| 03_Cheng AH_4 | 3.4% | 148.25 [128.01, 168.49] | 24 |
| 11_Huang PC_19 | 3.8% | 127.40 [111.35, 143.45] | 24.5 |
| 11_Huang PC_20 | 4.2% | 95.38 [83.92, 106.84] | 24.5 |
| 12_Yanez E_22 | 4.1% | 127.00 [113.79, 140.21] | 25 |
| 12_Yanez E_21 | 4.8% | 98.43 [94.40, 102.45] | 25.5 |
| 25_Egana JI_39 | | Not estimable | 26 |
| 25_Egana JI_38 | | Not estimable | 26 |
| R12_Vargas E_47 | 4.7% | 80.25 [75.88, 84.61] | 26.5 |
| 17_Dutra de Oliveira JE_29 | | Not estimable | 26.5 |
| R12_Vargas E_46 | 4.7% | 92.37 [87.07, 97.68] | 28 |
| 16_Agarwal KN_28 | 0.0% | 90.37 [82.11, 98.63] | 32 |
| 28_Campbell WW_41 | 3.5% | 92.97 [73.72, 112.23] | 33.5 |
| 20-2_Chen XC_32 | 4.3% | 147.67 [136.58, 158.76] | 42 |
| 03_Cheng AH_5 | 4.2% | 138.18 [126.09, 150.27] | 68 |
| 04_Uauy R_7 | 2.2% | 102.47 [69.16, 135.77] | 71 |
| 24_De Unamuno MRDL_37 | 0.0% | 113.13 [94.00, 132.26] | 71 |
| 28_Campbell WW_43 | 4.4% | 104.15 [93.99, 114.31] | 72 |
| **Total (95% CI)** | **100.0%** | **107.74 [100.99, 114.48]** | |

Heterogeneity: Tau² = 244.68; Chi² = 278.53, df = 24 (P < 0.00001); I² = 91%
Test for overall effect: Z = 31.30 (P < 0.00001)

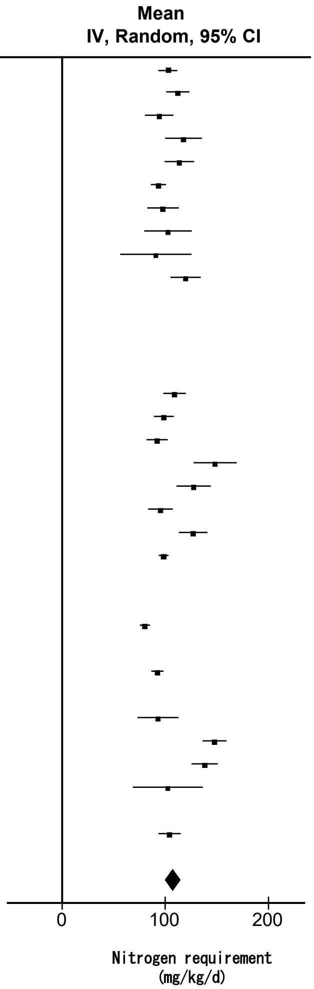

# Supplement Figure S1. H

## Male Tropical

| Study | Weight | Mean IV, Random, 95% CI | Age |
|---|---|---|---|
| 14_Scrimshaw NS_25 | 0.0% | 103.05 [94.58, 111.53] | 19 |
| 21_Young VR_33 | 0.0% | 112.21 [101.66, 122.76] | 19.4 |
| 13_Istfan N_23 | 0.0% | 94.19 [80.92, 107.47] | 19.5 |
| 14_Scrimshaw NS_24 | 0.0% | 117.67 [100.66, 134.69] | 20 |
| 09_Bourges H_16 | 0.0% | 113.65 [99.81, 127.48] | 20.5 |
| 07_Inoue G_12 | 0.0% | 93.43 [86.67, 100.19] | 21 |
| 07_Inoue G_14 | 0.0% | 97.67 [83.02, 112.33] | 21 |
| 09_Bourges H_17 | 0.0% | 102.65 [80.27, 125.03] | 21 |
| 21_Young VR_34 | 0.0% | 90.94 [57.04, 124.84] | 21.1 |
| 07_Inoue G_13 | 0.0% | 119.77 [105.87, 133.67] | 22 |
| 06_Fajardo LF_10 | 10.5% | 133.07 [118.18, 147.95] | 22.5 |
| 06_Fajardo LF_11 | 11.1% | 134.35 [122.46, 146.25] | 23 |
| 08_Tontisirin K_15 | 11.4% | 138.10 [127.89, 148.31] | 23 |
| 29_Atinmo T_45 | 11.8% | 108.58 [101.45, 115.71] | 23 |
| 15_Vannucchi H_26 | 0.0% | 108.94 [98.72, 119.17] | 23 |
| 19_Ozalp I_31 | 0.0% | 98.54 [89.47, 107.60] | 23 |
| 01_Clark HE_2 | 0.0% | 92.18 [82.46, 101.90] | 24 |
| 03_Cheng AH_4 | 0.0% | 148.25 [128.01, 168.49] | 24 |
| 11_Huang PC_19 | 0.0% | 127.40 [111.35, 143.45] | 24.5 |
| 11_Huang PC_20 | 0.0% | 95.38 [83.92, 106.84] | 24.5 |
| 12_Yanez E_22 | 0.0% | 127.00 [113.79, 140.21] | 25 |
| 12_Yanez E_21 | 0.0% | 98.43 [94.40, 102.45] | 25.5 |
| 25_Egana JI_39 | 11.4% | 80.12 [70.32, 89.92] | 26 |
| 25_Egana JI_38 | 11.4% | 102.76 [93.03, 112.48] | 26 |
| R12_Vargas E_47 | 0.0% | 80.25 [75.88, 84.61] | 26.5 |
| 17_Dutra de Oliveira JE_29 | 11.1% | 115.27 [103.33, 127.21] | 26.5 |
| R12_Vargas E_46 | 0.0% | 92.37 [87.07, 97.68] | 28 |
| 16_Agarwal KN_28 | 11.6% | 90.37 [82.11, 98.63] | 32 |
| 28_Campbell WW_41 | 0.0% | 92.97 [73.72, 112.23] | 33.5 |
| 20-2_Chen XC_32 | 0.0% | 147.67 [136.58, 158.76] | 42 |
| 03_Cheng AH_5 | 0.0% | 138.18 [126.09, 150.27] | 68 |
| 04_Uauy R_7 | 0.0% | 102.47 [69.16, 135.77] | 71 |
| 24_De Unamuno MRDL_37 | 9.7% | 113.13 [94.00, 132.26] | 71 |
| 28_Campbell WW_43 | 0.0% | 104.15 [93.99, 114.31] | 72 |
| **Total (95% CI)** | **100.0%** | **112.52 [99.30, 125.74]** | |

Heterogeneity: Tau² = 373.48; Chi² = 114.40, df = 8 (P < 0.00001); I² = 93%
Test for overall effect: Z = 16.68 (P < 0.00001)

Mean
IV, Random, 95% CI

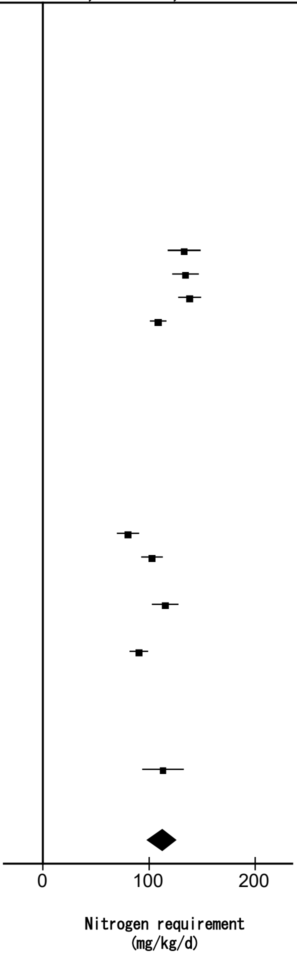

# Supplement Figure S1. I

## Female Temperate

| Study | Weight | Mean IV, Random, 95% CI | Age |
|---|---|---|---|
| 05_Thomas MR_8 | 13.3% | 95.39 [76.66, 114.11] | 20 |
| 02_Alford BB_3 | 16.1% | 123.73 [112.77, 134.69] | 21 |
| 23_Kaneko K_36 | 15.7% | 103.03 [90.92, 115.13] | 21 |
| 06_Fajardo LF_09 | 0.0% | 111.61 [108.27, 114.94] | 22.5 |
| 18_Hussein MA_30 | 0.0% | 85.96 [81.47, 90.46] | 22.5 |
| 26_Egun GN_40 | 0.0% | 81.38 [79.77, 82.98] | 23.5 |
| 10_Calloway DH_18 | 11.1% | 85.72 [60.95, 110.49] | 25 |
| R45_Pasricha S_48 | 0.0% | 107.51 [100.72, 114.30] | 26 |
| 16_Agarwal KN_27 | 0.0% | 104.83 [98.75, 110.91] | 32 |
| 28_Campbell WW_42 | 16.1% | 106.12 [95.03, 117.21] | 33.5 |
| 28_Campbell WW_44 | 16.7% | 87.59 [78.59, 96.59] | 72 |
| 04_Uauy R_6 | 10.9% | 145.35 [120.08, 170.62] | 74 |
| **Total (95% CI)** | **100.0%** | **105.97 [92.68, 119.26]** | |

Heterogeneity: Tau² = 254.16; Chi² = 38.53, df = 6 (P < 0.00001); I² = 84%
Test for overall effect: Z = 15.63 (P < 0.00001)

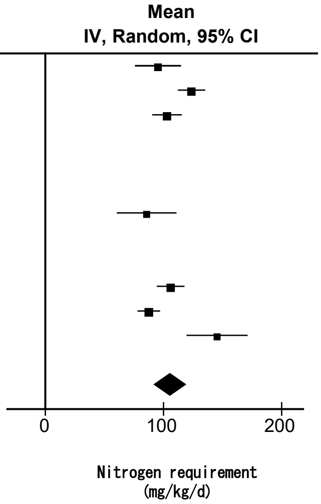

# Supplement Figure S1. J

## Female Tropical

| Study or Subgroup | Weight | Mean IV, Random, 95% CI | Age |
|---|---|---|---|
| 05_Thomas MR_8 | 0.0% | 95.39 [76.66, 114.11] | 20 |
| 02_Alford BB_3 | 0.0% | 123.73 [112.77, 134.69] | 21 |
| 23_Kaneko K_36 | 0.0% | 103.03 [90.92, 115.13] | 21 |
| 06_Fajardo LF_09 | 20.2% | 111.61 [108.27, 114.94] | 22.5 |
| 18_Hussein MA_30 | 20.1% | 85.96 [81.47, 90.46] | 22.5 |
| 26_Egun GN_40 | 20.4% | 81.38 [79.77, 82.98] | 23.5 |
| 10_Calloway DH_18 | 0.0% | 85.72 [60.95, 110.49] | 25 |
| R45_Pasricha S_48 | 19.6% | 107.51 [100.72, 114.30] | 26 |
| 16_Agarwal KN_27 | 19.8% | 104.83 [98.75, 110.91] | 32 |
| 28_Campbell WW_42 | 0.0% | 106.12 [95.03, 117.21] | 33.5 |
| 28_Campbell WW_44 | 0.0% | 87.59 [78.59, 96.59] | 72 |
| 04_Uauy R_6 | 0.0% | 145.35 [120.08, 170.62] | 74 |
| **Total (95% CI)** | **100.0%** | **98.16 [83.37, 112.95]** | |

Heterogeneity: Tau² = 278.64; Chi² = 319.50, df = 4 (P < 0.00001); I² = 99%
Test for overall effect: Z = 13.01 (P < 0.00001)

**Mean IV, Random, 95% CI**

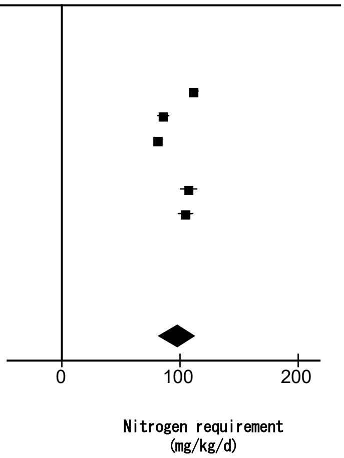

Supplement: Supplementary file 1 [file nutrients-17-02615-s001.zip › Supplementary_Figure_S1.pdf]
